# Metagenomics reveals that detoxification systems are underrepresented in marine bacterial communities

**DOI:** 10.1186/1471-2164-15-749

**Published:** 2014-09-01

**Authors:** Johan Bengtsson-Palme, Magnus Alm Rosenblad, Mikael Molin, Anders Blomberg

**Affiliations:** Department of Chemistry and Molecular Biology, University of Gothenburg, Box 462, SE-405 30 Göteborg, Sweden; Department of Infectious Diseases, Institute of Biomedicine, the Sahlgrenska Academy, University of Gothenburg, Guldhedsgatan 10, SE-413 46 Göteborg, Sweden

**Keywords:** Detoxification, Ecotoxicology, Global ocean sampling, Marine, Metagenomics, Oxidative stress, Toxic metals

## Abstract

**Background:**

Environmental shotgun sequencing (metagenomics) provides a new way to study communities in microbial ecology. We here use sequence data from the Global Ocean Sampling (GOS) expedition to investigate toxicant selection pressures revealed by the presence of detoxification genes in marine bacteria. To capture a broad range of potential toxicants we selected detoxification protein families representing systems protecting microorganisms from a variety of stressors, such as metals, organic compounds, antibiotics and oxygen radicals.

**Results:**

Using a bioinformatics procedure based on comparative analysis to finished bacterial genomes we found that the amount of detoxification genes present in marine microorganisms seems surprisingly small. The underrepresentation is particularly evident for toxicant transporters and proteins involved in detoxifying metals. Exceptions are enzymes involved in oxidative stress defense where peroxidase enzymes are more abundant in marine bacteria compared to bacteria in general. In contrast, catalases are almost completely absent from the open ocean environment, suggesting that peroxidases and peroxiredoxins constitute a core line of defense against reactive oxygen species (ROS) in the marine milieu.

**Conclusions:**

We found no indication that detoxification systems would be generally more abundant close to the coast compared to the open ocean. On the contrary, for several of the protein families that displayed a significant geographical distribution, like peroxidase, penicillin binding transpeptidase and divalent ion transport protein, the open ocean samples showed the highest abundance. Along the same lines, the abundance of most detoxification proteins did not increase with estimated pollution. The low level of detoxification systems in marine bacteria indicate that the majority of marine bacteria have a low capacity to adapt to increased pollution. Our study exemplifies the use of metagenomics data in ecotoxicology, and in particular how anthropogenic consequences on life in the sea can be examined.

**Electronic supplementary material:**

The online version of this article (doi:10.1186/1471-2164-15-749) contains supplementary material, which is available to authorized users.

## Background

Prokaryotes have evolved a rich repertoire of detoxification mechanisms that enable them to exploit and be successful in highly diverse ecological niches. By this repertoire bacteria impact the environment since microbial communities alter bioavailability of toxic metals, such as arsenic and selenium [1], and degrade a large array of xenobiotic compounds [2]. In habitats under anthropogenic influence, where a large variety of toxic substances can be present, appropriate detoxification is believed to be instrumental for survival and growth. Thus, detoxification proteins are likely to play a major part in natural ecosystem processes, and in a broader perspective also contribute to human health and society. Despite their importance, there is a lack in our understanding of the distribution and use of these detoxification systems in natural environments, like the ocean. Metagenomics, i.e. the sequencing of DNA isolated from environmental samples, provides a new way to study communities in microbial ecology, and enables studies of detoxification systems in natural environments in an unbiased way and on a genome-wide scale. The gene-centered approach to ecotoxicology will become increasingly important since sequencing of microbial communities can provide relationships between detoxification systems and selective pressures in specific environments, indicating how various selection pressures shape the gene content of microbial organisms.

Metagenomics fosters studies of the unknown diversity, as most of the microbes in nature cannot be cultured in the laboratory [3]. This novel strategy has successfully been applied to assess a variety of biological questions, revealing, for example, a wide extension of the bacterial kinome [4], functional novelties in light-mediated pathways [5], the distribution of photosynthetic light-harvesting genes in phytoplankton communities [6], and shedding light on the ecological roles of proteins with unknown functions [7]. However, metagenomics also provides a means to assess how particular selection pressures affect the gene content of a certain environment and, conversely, what selection pressures that are disclosed by the gene content in an environmental sample. For example, methane consumption has been correlated to the presence of methane degrading enzymes, like methane monooxygenases, by metagenomics on thawing samples from the permafrost [8]. Also, in a highly contaminated river in India, large numbers of resistance genes could be found as a consequence of up-stream antibiotic pollution from factories [9]. Furthermore, in ground water highly contaminated with heavy metals, nitric acid and organic solvents, enhanced abundance of resistance genes towards e.g. nitrate, cadmium and acetone has been reported [10], and quaternary ammonium compound exposure can cause enrichment of efflux pumps and cell envelope modification systems in microbial communities [11]. Thus, screening for genes involved in the handling of xenobiotics in environmental sequence data could provide further understanding of how microbes cope with high levels of toxicants, and aid our search for biotechnologically important detoxification genes. In this work, we have used sequence data from the Global Ocean Sampling (GOS) expedition to investigate toxicant selection pressures revealed by the presence of functionally characterized detoxification genes in marine environments. The GOS data still constitutes the largest ocean study performed over a geographically wide area in a consistent manner.

Protein sequences can be grouped by functional criteria and there are currently around 15,000 – 17,000 classified protein families [12, 13]. Here we systematically analyze a subset of those that are linked to detoxification. We thereby provide evidence for the extent to which toxicants in the marine milieu affect microorganisms and whether the genes present indicate differences in toxicant selection pressures between marine environments, like the open ocean compared to coastal waters. To be able to efficiently capture the broad range of potential toxicants, and to address differences between various sampling sites, we have selected well-characterized detoxification protein families representing biological systems protecting microorganisms from a variety of stressors, such as metals, organic compounds, antibiotics and oxygen radicals. We find that the amount of detoxification genes present in marine microorganisms seems to be surprisingly small. This is particularly evident for toxicant transporters, as well as for protein families detoxifying metals. Exceptions are enzymes involved in the oxidative stress defense, where we found that peroxidase enzymes are more abundant than expected. In contrast, catalases are almost completely absent from the marine environment, suggesting that peroxidases and peroxiredoxins constitute a core line of defense against reactive oxygen species (ROS) in this milieu.

## Results

### Selection of well-characterized bacterial systems directly linked to detoxification

To enable a wide and unbiased detoxification scope in our metagenomics analysis we initially selected all proteins in the NCBI database related to detoxification mechanisms, based on their Gene Ontology (GO) classifications [14]. The term detoxification itself is not part of the GO terminology, and thus we used nine rather wide GO terms with links to detoxification, e.g. response to toxin (0009636), response to oxidative stress (0006979) and response to xenobiotic stimulus (0009410) (see Additional file 1: Table S1 for the complete list of GO terms used). In addition, we initially restricted our analysis to functionally well-characterized detoxification systems encountered in the model bacterium *Escherichia coli* for the following reasons; i) to focus on detoxification systems in bacteria (which is most adequate for the GOS data), ii) to avoid any misclassification and/or uncertainties about the proteins' functional roles, and iii) to obtain a wide set of detoxification genes, since *E. coli* has a comparably large genome and thus harbors a rather wide array of detoxification systems. All obtained detoxification-linked protein sequences were matched against the Pfam database of profile-HMMs using HMMER [15], which resulted in a list of 159 Pfam protein profiles (Additional file 2: Table S2). The use of Pfam profile-HMMs representing the protein families in our searches of the metagenomic data allows us to find protein sequences from a broad range of species, due to the Hidden Markov Models’ high sensitivity with regards to amino acid changes also over long evolutionary distances [16–18]. We finally manually removed protein families solely indirectly classified as linked to detoxification e.g. ribosomal proteins and tRNA synthetases. In this way, we reduced the list to 31 strictly detoxification-related protein families with profile-HMMs, mainly representing metal resistance, toxin transporters and oxidative stress protection (Table 1).

**Table 1:** **Detoxification protein families investigated in this work, divided into functional categories**

Protein family	Pfam name	Number of reads GOS Initial	Number of reads GOS Reciprocal	GOS Reciprocal (%)	Genomes average (genes/genome)	Genomes CV (%)
**Metal resistance**						
Arsenate reductase	ArsC	993	853	85.9	1.1	108
Divalent ion tolerance protein	CutA1	511	511	100	0.4	127
Copper resistance protein CopC	CopC	472	426	90.3	0.3	206
Multicopper oxidase	Cu-oxidase_3	435	313	72	0.7	154
Copper transporter	CutC	258	258	100	0.2	182
Tellurite resistance protein	TehB	2956	244	8.3	0.2	206
Arsenical pump membrane protein	ArsB	177	19	10.7	0.3	218
Cadmium binding protein	YodA	1	0	0	0.0	492
**Transporters**						
Major facilitator family	MFS_1	22627	19301	85.3	23.1	108
ACR transporter	ACR_tran	10591	10337	97.6	4.4	126
Multi antimicrobial extrusion protein	MatE	5164	4308	83.4	2.2	120
Efflux pump	HlyD	4048	2693	66.5	6.9	121
Small Multidrug Resistance protein	Multi_Drug_Res	2194	2097	95.6	1.0	134
C4-dicarboxylate transporter	C4dic_mal_tran	71	71	100	0.3	173
**Oxidative stress**						
Peroxiredoxin	AhpC-TSA	14009	7397	52.8	3.5	91
Thioredoxin	Thioredoxin	8221	4668	56.8	2.7	67
Redoxin	Redoxin	13178	4410	33.5	1.7	108
Peroxidase	peroxidase	3067	3065	99.9	0.4	154
Glutathione peroxidase	GSHPx	2380	2218	93.2	0.6	131
Superoxide dismutase	Sod_Fe_C	935	796	85.1	0.9	78
Catalase	Catalase	91	88	96.7	0.8	146
**Other detoxification systems**						
Bacitracin resistance protein	BacA	2803	2799	99.9	0.8	73
Organic solvent tolerance protein	OstA_C	1094	1086	99.3	0.4	121
Fusaric acid resistance protein	FUSC	67	65	97	0.8	205
Di-haem cytochrome c peroxidase	CCP_MauG	606	582	96	0.5	211
Beta-lactamase	Beta-lactamase	5196	4985	95.9	2.6	149
NADH oxidase	Oxidored_FMN	2598	2349	90.4	1.9	130
Penicillin binding transpeptidase	Transpeptidase	9702	8379	86.4	2.1	72
Multiple antibiotic resistance operon repressor	MarR	2949	2078	70.5	5.8	128
MAATS-type multidrug transcriptional repressor	TetR_C_2	21	12	57.1	0.1	343
NADPH-dependent FMN reductase	FMN_red	2222	1076	48.4	2.5	110
**Control protein families**						
Elongation factor TS	EF_TS	2780	2734	98.3	0.8	49
DNA polymerase A	DNA_pol_A	5372	4973	92.6	0.9	55
RNA polymerase rpoB	RNA_pol_Rpb2_6	5847	5106	87.3	1.0	9

### Initial analysis of the abundance of detoxification systems in the Global Ocean Sampling (GOS) data

The 31 Pfam profile-HMMs for detoxification proteins were compared to the 6,028,191 proteins in the GOS data set [19] using hmmsearch, which is part of the HMMER3 software [15]. The resulting number of sequences found in this initial search is indicated under ”GOS Initial” in Table 1. The most abundant (in terms of the number of genomic reads) of all the detoxification proteins were the major facilitator family of transport proteins (22,627 reads) and peroxiredoxins, involved in oxidative stress defense (14,009 reads).

To rule out uncertainties in protein classifications, matches were in a second step scanned against the entire Pfam profile database and only reciprocal hits, i.e. sequences that best matched the same Pfam profile as initially found them, were further analyzed (”GOS Reciprocal” in Table 1). Note that in certain cases the GOS initial and the GOS reciprocal values differ quite substantially. This is particularly pronounced for the peroxiredoxin, thioredoxin and redoxin families where the reciprocal matches were below 60% of their initial numbers. These larger discrepancies are almost exclusively due to hits to protein families belonging to the same Pfam clan and therefore showing fairly high sequence similarity, which is known to be the case for e.g. the peroxiredoxin/thioredoxin proteins [20]. For the reciprocal matches, the major facilitator family and the ACR transporters were the most abundant protein classes, followed by penicillin binding transpeptidase and peroxiredoxin.

### Marine bacteria harbor a low number of detoxification systems

The abundances of various detoxification systems in an environmental sample may tell something about the selection pressure in that particular environment. The statistical approaches proposed for analyzing these data usually test the null hypothesis that the abundances of genes are equal across samples [21–23]. However, some genes are frequently present as larger paralogous families in microbes, and thus blindly applying the reciprocal GOS hits indicated in Table 1 would result in a misinterpretation of the selection pressure for certain detoxification systems in that particular environment. To circumvent this bias in our analyses we employed a procedure where we normalize for the extent that detoxification systems are generally found in typical bacterial genomes.

In order to enable this type of genome content normalization, we used the same 31 Pfam profiles and the reciprocal search procedure to determine the number of genes belonging to our detoxification set in 835 fully sequenced and annotated bacterial genomes (Additional file 3). It is clear from this analysis that the number of genes corresponding to each detoxification protein family, in each bacterial genome, exhibits rather great variation between bacterial species (Table 1; Additional file 4: Table S3; Additional file 5: Figure S1). The high sensitivity of the Pfam profiles was clearly apparent since we picked up homologous proteins at very wide evolutionary distances, e.g. the divalent ion tolerance protein CutA1 was found in the archaeon *Methanococcus maripaludis* as well in the eubacteria *Burkholderia mallei* and *Prochlorococcus marinus*. The majority of bacterial species contain a wide spectrum of detoxification systems, where classes like major facilitator transporters (genome average 23.1 genes), ACR transporters (genome average 4.4 genes) and thioredoxin (genome average 2.7 genes) are present in multiple copies in almost all genomes. However, it was also clear that some species apparently lack even these common detoxification systems, e.g. the parasitic microbes *Chlamydia trachomatis* and *Mycobacterium tuberculosis*, and the coefficients of variation (CV) were rather large (above 100%) for many of the protein families. This was most apparent for genes where the average was below 1 and where many genomes in fact lack the gene completely, like copper resistance protein CopC and tellurite resistance protein TehB which both exhibited CVs that were greater than 200% and with averages around 0.2-0.3 genes per genome. In fact, the majority of detoxification families had average and median values of gene copies below 1, revealing that these genes are missing in many genomes. The overall correlation between the average and median values was high (R^2^ = 0.96; Additional file 6: Figure S2) and all analyses below have been performed using either of the two, giving similar results (data not shown).

**Figure 1:**
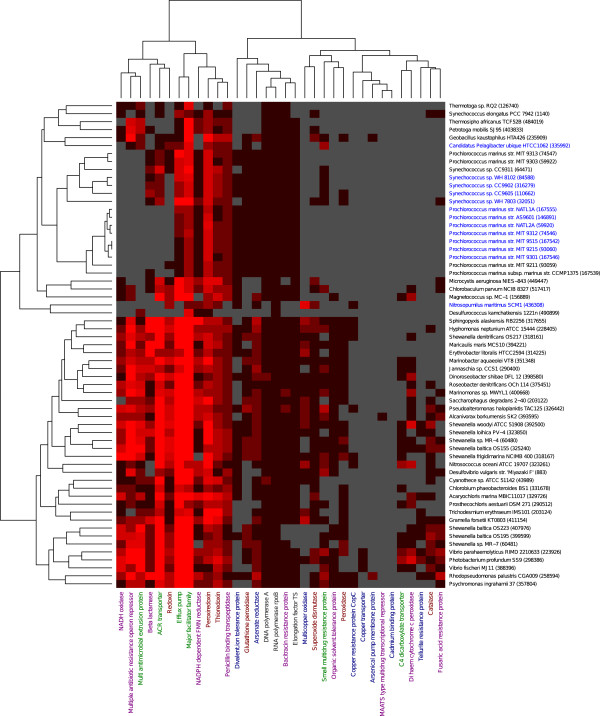
**Presence of detoxification proteins in genomes of various marine bacteria.** The Pfam profiles for all studied detoxification proteins were screened against 835 fully sequenced bacterial genomes. The data for the subset of bacterial species/strain that have been reported as marine are on display. The most commonly found marine bacteria, based on fragment recruitment to genome reference sequences [24], are indicated in blue. No gene found (grey), 1 gene per genome (black) and greater than 1 gene per genome (red).

When we exclusively examined the genomes of marine bacteria (61 species/strains fully sequenced; Figure 1)*,* we found that the most common marine bacteria in the GOS data (based on read recruitment to genome reference sequences [24]), like *Prochlorococcus* and *Synechococcus*, have substantially fewer detoxification genes than the less common marine species. Two exceptions were the glutathione peroxidase and peroxiredoxin families, of which these marine bacteria have higher numbers than the average across all sequenced bacteria in our set (Additional file 4: Table S3). Furthermore, the streamlined genome of the very common marine bacteria *Candidatus Pelagibacter ubique* [25], contained few of the investigated detoxification genes, as would be expected from its small genome size. Surprisingly however, the number of detoxification systems in the larger genomes of *Prochlorococcus* and *Synechococcus* were also very low and correspond very closely to the numbers in *Candidatus Pelagibacter*.

### RNA polymerase *rpoB*provides a good proxy for the number of genomes in the sample

In order to set our data in relation to the number of genomes present we included in our analysis three control proteins with no clear link to detoxification but part of central cellular functions; RNA polymerase Rpb2 domain 6 (corresponding to the *rpoB* gene), DNA polymerase A and translational elongation factor TS. The control genes were chosen to represent genes generally present in bacteria and mostly in one copy per bacterial genome (Table 1 and Additional file 4: Table S3). We observed that for our three control proteins, the RNA polymerase gene was most consistently found in one copy per genome for the fully sequenced bacteria and displayed the lowest variation (1.0 ± 0.1 [±SD]). This consistency also holds if only marine genomes, or only the most abundant bacterial species in the ocean, are considered (Additional file 4: Table S3). In addition, RNA polymerase appeared quite evenly distributed at all the different GOS sites and consequently exhibited a low coefficient of variation (25%). Hence, we have used the RNA polymerase gene to normalize the GOS data to the expected number of genomes per sample.

### Most detoxification proteins are under-represented at the GOS sites in relation to the expectation

**Figure 2:**
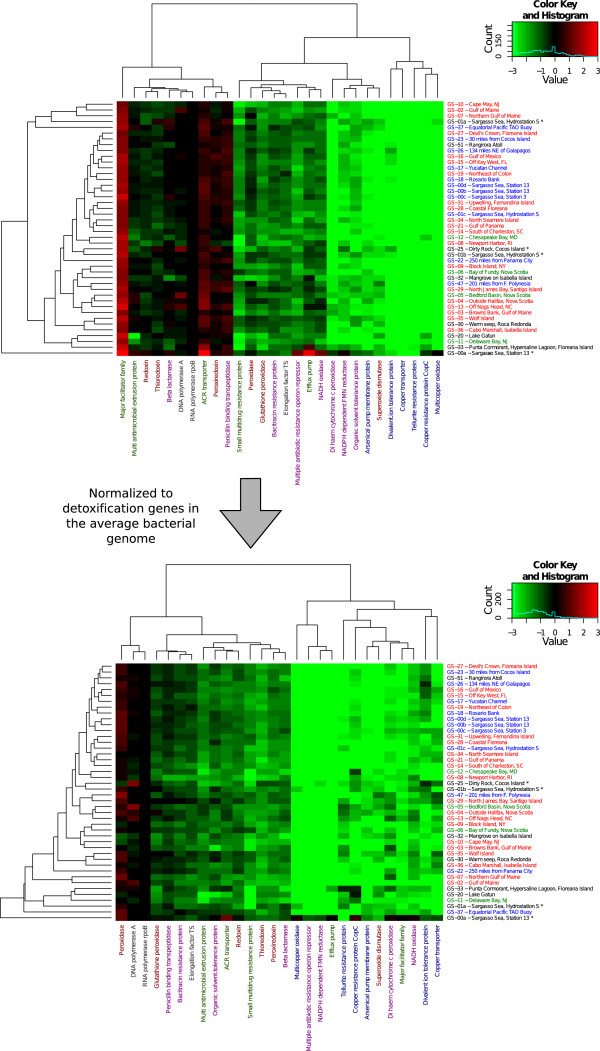
**Distribution of detoxification protein families in the GOS data.** Gene counts normalized to per genome-equivalents based on the occurrence of the RNA-polymerase gene (top), and gene counts normalized to the average detoxification gene content of 835 completely sequenced bacterial genomes (bottom). For site names, blue color indicates open ocean sites, red corresponds to coastal sites, and green represents estuaries and embayments. Protein families are color coded as follows; red – oxidative stress, blue – metal resistance, green – transporters, purple – other detoxification systems, grey – control proteins.

Two-dimensional hierarchical clustering was initially performed, after normalization to the number of genomes (based on the RNA polymerase data), to visualize relations between detoxification systems as well as between sampling sites (Figure 2*,* top). Interestingly, we found that almost all investigated detoxification protein families are on average found less than once per genome in the GOS data (Figure 2, top), i.e. these genes are missing in a large portion of the marine bacterial genomes. The exceptions were the major facilitator transporter, the ACR transporter, the penicillin binding transpeptidase, and the peroxiredoxin families, that were mostly represented by one or more genes per genome.

However, since some of these detoxification systems are part of commonly found paralogous families, we also compared our findings in the GOS data to what would be expected for the various detoxification systems in an average bacterial genome (using our data on the 835 fully sequenced genomes). Interestingly, after this “paralog normalization” virtually all detoxification protein families were still underrepresented at the various GOS sites (Figure 2, bottom; Additional file 7: Figure S3). Furthermore, we found that the detoxification systems were distributed in two distinct groups. For example, all the metal resistance proteins, such as copper transporters and tellurite resistance proteins, belonged to a cluster containing markedly underrepresented detoxification components in marine bacteria (GOS) compared to an average bacterial genome. The second group included all investigated proteins related to the oxidative stress response, except for the superoxide dismutase family. Interestingly, we also found that in the most frequently encountered marine species (based on read recruitment [24], i.e. the genera *Pelagibacter*, *Synechococcus, Prochlorococcus* and *Nitrosopumilus*) there is a marked underrepresentation of certain detoxification systems (Figure 1). We conclude that in a majority of cases, abundance of the detoxification genes did not correlate with ecological success.

### Analysis of marine non-*E. coli*detoxification systems

**Figure 3:**
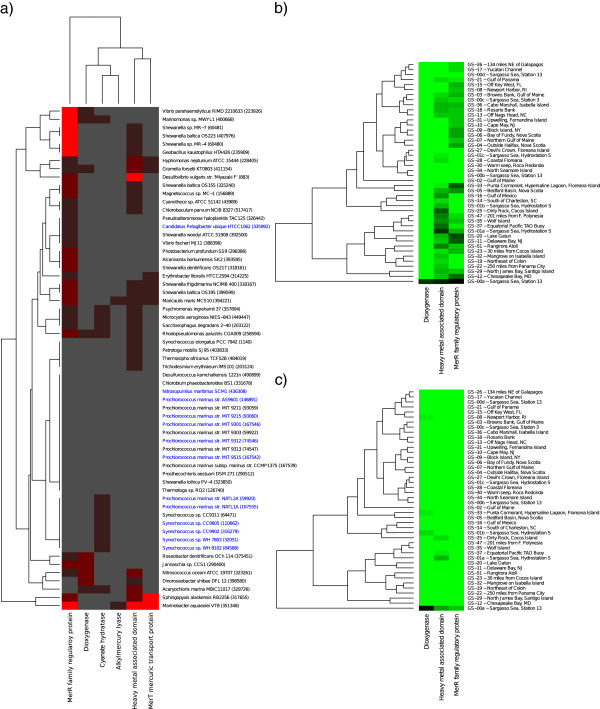
**Occurrence and distribution of six additional protein families in marine genomes and the GOS data.** Occurrence of additional detoxification protein families not present in *E. coli* in fully sequenced and annotated marine genomes **(a)**, their distribution in the GOS data normalized to per-genome equivalents **(b)**, and normalized to the average detoxification gene content of 835 completely sequenced bacterial genomes **(c)**. Bacterial names in blue color indicate species that are among the most commonly found in marine environments.

To make sure we provide an exhaustive description of detoxification, we next extended our analysis to include six additional protein families, known to be involved in detoxification in marine bacteria [26–29], but not present in *E. coli* (Additional file 8: Table S4). Investigation of these additional detoxification families revealed very similar results to the findings based on our initial set of 31 well-characterized *E. coli*-derived Pfam families: i) all six protein families were lowly represented in the GOS data (Additional file 8: Table S4), as well as in marine genomes (Figure 3a), ii) those that were abundant enough to generate distribution data, all showed up in less than one copy per genome at virtually all sites (Figure 3b), and iii) when compared to what would be expected from the sequenced bacterial genomes, these protein families showed the same pattern of marked underrepresentation (Figure 3c). We conclude that all data taken together strongly indicates that our results of general underrepresentation in marine bacteria will hold for most detoxification-related protein families.

### Geographical implications and anthropogenic influence

Our initial hypothesis was that marine sampling sites close to the coast would be more exposed to anthropogenic influence. Thus, we expected a greater repertoire and/or abundance of detoxification systems at these sites compared to sites from the open ocean. The GOS sampling sites are located in the Atlantic and the Pacific Oceans, and include samples taken from open ocean, coastal and estuarine habitats [30, 31].

When normalized to the number of genes present in the average bacterial genome (Figure 2, bottom; note that the different sampling sites are color coded by geographical location), sites expected to have similar environmental conditions mostly clustered together based on their content of detoxification genes. For example, the three Sargasso Sea samples GS-00b, c and d clustered tightly together. However, not all open ocean environments clustered nicely with the Sargasso Sea samples. For example, the sites GS-22 and GS-37 seem to be atypical from the other open ocean samples, indicating that these sites have a different composition of detoxification proteins. Two of the most atypical environments, the high saline (GS-33) and fresh water (GS-20) samples, clustered separately from most of the other samples, while the two samples filtered to contain slightly larger microorganisms in the 0.8 to 3.0 μm range (GS-01b and GS-25) also clustered together. The latter two samples are expected to contain small single-cell eukaryotes as well as bacteria with large cell sizes. Similarly, the GS-00a site, that have been reported to be contaminated by *Burkholderia* [32] clustered separately from all other samples. We conclude that geographical location seemed to have an influence on the distribution of some, but far from all, of the detected detoxification systems in the metagenomic dataset.

**Figure 4:**
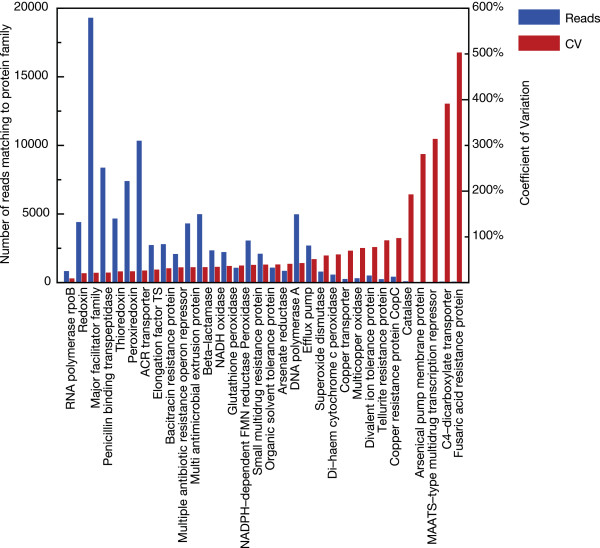
**Variability of the detoxification proteins at the investigated marine sites.** Variance expressed as coefficient of variation (CV) over all the sampled sites (right y-axis) in relation to the average number of reads belonging to that protein family (left y-axis).

It was also clear from the clustering that certain detoxification proteins were rather evenly distributed, while the appearance of others differed quite substantially between sites. This was in particular apparent when considering the overall variance over sampling sites for each protein (Figure 4); e.g. redoxin and the penicillin binding transpeptidase were roughly equally present at all sites (CV < 50%), while proteins like the C4-dicarboxylate transporter and arsenical pump membrane protein displayed a drastic variation in abundance depending on the sampling site (CV > 200%). However, for the highly variable proteins we also found a strong correlation to low abundance, indicating that for these proteins stochasticity plays a greater role and that the quantitative data from these sites should be viewed with caution. However, the majority of detoxification proteins displayed low CV values in the range 25 – 75% over the sampling sites, not exhibiting markedly greater variability than we observed for the three control proteins (RNA polymerase CV 25%; elongation factor CV 30%; DNA polymerase CV 50%).

**Figure 5:**
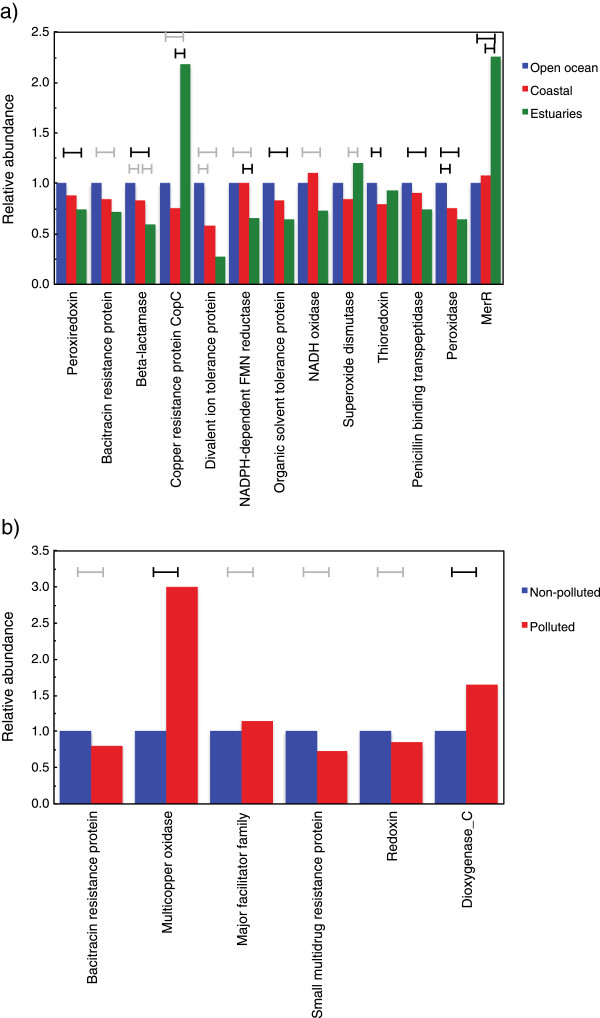
**Abundance of detoxification proteins between marine habitats. a)** Comparison of the relative abundance of the detoxification proteins between the three major habitats open ocean (blue bars), coastal (red bars) and estuary (green bars). **b)** Comparison of relative abundance between non-polluted (blue) and polluted (red) sites. Bars indicate relative abundance in relation to the protein family abundance in the open ocean sites **(a)** or non-polluted sites **(b)**, fixed to one, and only proteins that exhibited a significant difference in relative abundance between sites are displayed. Brackets indicate which comparisons that were significant; black brackets (p < 0.01) and grey brackets (p < 0.05).

When we extended this analysis to compare the different habitats (open ocean, coastal and estuaries), we found that some of the detoxification systems were significantly more abundant at certain types of habitats, e.g. the beta-lactamase and the penicillin binding transpeptidase families were both more abundant in the open ocean samples compared to the estuaries (p < 0.01; Figure 5a). Similarly, the peroxidases and thioredoxins were more common in the open ocean compared to coastal waters (p < 0.05). In addition, when we analyzed the detoxification genes in the three functional categories metal resistance, transporters and oxidative stress together, it was apparent that oxidative stress genes were significantly more present in open ocean versus either coastal or estuary (p < 0.01 in both cases, data not shown). It is clear from these analyses, that some detoxification systems were not equally distributed among marine sampling sites. However, we noted that the uneven distribution of some detoxification families was contrary to our initial expectation; in most cases the open ocean samples contained significantly higher levels of these detoxification proteins than the sites closer to land. Nevertheless, two metal resistance-related proteins, copper resistance protein CopC and the mercury resistance operon regulator MerR, showed significantly increased abundances in estuarine environments where we would expect the influence from human activity to be the greatest (Figure 5a).

To further scrutinize the environmental distributions of detoxification systems, we used the geographical division based on estimated chemical composition at the various GOS sites proposed by Patel *et al.* (North Atlantic, Mid-Atlantic and Pacific samples) [33], and performed the same statistical analysis of relative abundance of detoxification proteins as for habitats (Additional file 9: Figure S4). However, it should be noted that estimated chemical information is obtainable for only 29 of the GOS sites, excluding all the estuarine sites, which might be most severely impacted by human activities. Here, the divalent ion tolerance protein stood out as around four times more common in Mid-Atlantic and Pacific than in North Atlantic samples, and the oxidative stress response proteins peroxiredoxin and redoxin were more prevalent in Pacific samples than in the North Atlantic (p < 0.01 in all cases). In general, proteins that exhibited significant differences between North Atlantic, Mid-Atlantic and Pacific locations were more abundant in the Pacific and Mid-Atlantic samples, than in the North Atlantic. The notable exception to this was the multicopper oxidase family, which was significantly more abundant in both the Atlantic samples than in the Pacific (p < 0.01).

The Patel *et al.* study also estimated the anthropogenic load on 29 of the samples investigated in our study [33]. We used their additional environmental variables together with the metadata from the GOS samples to make a principal component analysis (Additional file 10: Figure S5). This analysis revealed that most detoxification proteins did not correlate with pollution and shipping, but rather with physical properties such as temperature, salinity, water depth, and oxygen utilization. Finally, we separated the data into two sets, based on the estimated pollution at each site, producing a polluted and a non-polluted set of sampling sites (Additional file 11: Figure S6). It should be noted that estimated pollution correlated with shipping, but that high shipping levels did not always imply high pollution, and vice versa. Comparing the polluted and the non-polluted groups showed that proteins belonging to the multicopper oxidase and dioxygenase C families were significantly (p < 0.01) more abundant at polluted sites (Figure 5b).

## Discussion

### Underrepresentation of well-characterized detoxification genes in marine environments

The most striking finding of our metagenomic analysis of ocean samples is that there are surprisingly few detoxification genes present in bacteria living in the marine environment compared to what is present in bacterial genomes in general. This statement is supported by several observations. i) The overall level of detoxification genes in sequenced genomes of marine bacteria compared to general bacterial genomes is low. This is particularly evident for several general toxicant transporters, such as the ACR transporters and the major facilitator superfamily (Figure 1). In fact, in many of the marine bacteria most of the detoxification genes are missing completely. ii) The most abundant marine bacteria, and thus the ones clearly successful in the marine milieu, are missing a majority of the detoxification proteins. iii) There is a low level of detoxification genes in the GOS metagenomic data, regardless of sampling site. The overall low number of detoxification systems is consistent with findings that most marine bacteria lack a number of biological systems that are important or even essential in other environments [24]. These observations have important implications for how we view detoxification proteins in general and their involvement in shaping bacterial ecology in particular.

Many detoxification systems are part of a general core-set of genes that are present in most types of microorganisms, and high representation of those should not immediately be viewed as their level of importance for detoxification. To handle this situation, we have normalized the data to the number of gene copies expected to be found in general bacterial genomes. Detoxification genes are frequently present in multiple copies in microbial genomes, where gene multiplications are selected to yield high expression and/or functionally distinct paralogs to optimize survival and growth under harsh conditions. There are several examples of this, e.g. the drastically elevated copper tolerance of the European and Sake lineages of industrial yeast strains is related to high copy number for the gene encoding the copper binding protein Cup1 [34], and cadmium tolerance in the cyanobacterium *Synechococcus* has been linked to gene amplification of the metallothionein smt [35]. Thus, it is currently widely accepted that gene duplication is one of the main genetic mechanisms shaping organisms during long-term adaptation. Our “paralog normalization” for gene content per genome should emphasize decreases and increases in gene number in relation to the expectations.

It is interesting that we consistently find so low numbers of most detoxification proteins in the GOS data. This finding might seem contradictory to previous research on e.g. *Alteromonas macleodii*, showing presence of heavy metal resistance genes [26]. However, the *Alteromonas* genus is not frequently found in open ocean surface water samples that are predominant in the GOS data set [24]. This means that the fact that species of this genus and other less common genera possess heavy metal resistance genes will not be contributing enough to the overall abundance of these genes at any specific GOS site. If a variety of such rare opportunists such as *Alteromonas macleodii* [36] are present in surface water, we would expect to find small numbers of a wide range of detoxification genes, which is indeed the case in the GOS samples (Figure 2). Additionally, the detoxification genes in *Alteromonas* often seem to be located in genomic islands [26, 36], indicating that they might be part of mobile elements. This raises the possibility that these genes are lowly abundant under normal conditions, but in certain circumstances they might be selected for, spread through a population, and consequently show a temporal and/or spatial burst in distribution.

A strong contributing factor to the overall low abundance of detoxification genes at GOS sites is the high presence of *Candidatus Pelagibacter* species, which can constitute around a quarter of the bacteria present in ocean surface water [37]. In line with the extreme streamlining of the genomes in this genus [25], we find that most detoxification systems are lowly abundant, or even completely absent in the *Candidatus Pelagibacter ubique* genome (Figure 1). The same pattern repeats also for the cyanobacterial genera *Prochlorococcus* and *Synechococcus*, which also possess streamlined genomes containing few detoxification genes – although to a lower degree than *Pelagibacter*.

From our results, it is evident that we should not expect the genomes of abundant marine bacteria that have not yet been sequenced to contain a wide arsenal of detoxification genes, as a wide distribution of such microbes would have augmented the relative abundances of detoxification protein families far more than they are increased when normalized against marine bacteria in this study (Additional file 7: Figure S3). However, as there are still small numbers of many of those genes present in the GOS samples (Table 1), we would expect lowly abundant marine bacteria to harbor at least some of those families.

We cannot exclude that there could be novel systems for detoxification in the ocean not present and/or not characterized in *E. coli.* Groups of proteins with low sequence similarity have been shown to still fold in similar three-dimensional conformations and to exhibit the same enzymatic activity [38]. This stresses the importance of functional metagenomics studies, where libraries of natural DNA are screened for specific enzymatic activities. Such analyses for novel sequence-function relations are important to perform on naturally occurring marine microbes, to get a complete understanding of the detoxification capacity/potential in the ocean.

### Oxidative stress – catalases and peroxidases in marine bacteria

One important group of detoxification genes in the GOS data, which were found about as often as expected (compared to what is present in sequenced bacterial genomes), consisted of protein families involved in the oxidative stress response e.g. thioredoxins, glutathione peroxidases and peroxiredoxins (Figure 2; bottom). These proteins are also, in addition to being part of the oxidative stress defense, important in other aspects of cell physiology besides detoxification. In addition, it is rational that these genes are abundant in marine environments, as many of the most common microorganisms in the ocean are photosynthetic and produce oxygen and oxygen radicals requiring efficient detoxification systems [39]. An interesting aspect of the oxidative stress proteins analyzed is that the catalase family seems to be almost absent in marine environments (Table 1), while we found 1.6 times as many peroxidases than expected based on the sequenced genomes (Additional file 7: Figure S3). This indicates that protection against H_2_O_2_ could be conferred by peroxidases instead of catalases in dominant marine bacteria, as previously suggested by Bernroitner *et al.* for the cyanobacteria *Synechococcus* and *Synechocystis* [40]. The absence of typical metal-dependent catalases (katEs) in cyanobacteria has been proposed to reflect the absence of the need for diversification of catalases that coincided with the evolution of both eukaryotes and pathogenic bacteria, presumably to handle the high levels of H_2_O_2_ produced by host defences [41]. In this model, bifunctional catalase-peroxidases (katGs) as well as peroxidases and peroxiredoxins would provide sufficient protection against oxygen radicals produced upon photosynthesis. In fact, katGs represent an evolutionary ancient protein family that lies at the evolutionary origin of a major superfamily of peroxidases and are thought to have been present in cyanobacteria already before the evolution of oxygenic photosynthesis. In contrast to the GOS data, only two of the genomes of the most common marine bacteria contain peroxidases. A closer look at other peroxidase-active enzyme families, however, reveals that these marine bacteria contain more peroxiredoxins and glutathione peroxidases compared to the predicted “standard” bacterial genome (Additional file 4: Table S3). This suggests that enzymes other than typical metal-dependent catalases protect cells from H_2_O_2_ in the marine environment. Thus we conclude that peroxidases and/or peroxiredoxins appear to constitute the core cellular ROS defense mechanisms in the marine environment.

### The ecotoxicology of detoxification systems

We found our original hypothesis – that detoxification systems would generally be more abundant close to the coast – not to hold, except in three cases; copper resistance protein CopC, mercury resistance operon repressor MerR and superoxide dismutase, which were significantly more common in estuaries (p < 0.05). On the contrary, for most of the proteins that displayed a significant geographical distribution, like peroxidase, penicillin binding transpeptidase and divalent ion transport protein, the open ocean samples showed significantly higher abundance (Figure 5a). This is counter-intuitive since human activity should have a higher impact on waters close to the coast. The estimated anthropogenic load [33] was, unfortunately, not available for any of the estuarine samples. However, salinity can be used as a proxy for the impact from river outflow of fresh water into the sea. The coastal sites clearly exhibit a higher impact from fresh water rivers indicated by its substantially lower salinities (average salinity coastal 32.0‰ (±2.8), and open ocean 35.7‰ (±1.9)), even if there is generally quite some distance to land for the coastal sites. Thus, we would expect the coastal sites to be more polluted compared to samples from the open ocean. However, the chemical data for open ocean and coastal samples by Patel *et al.*, [33] indicates that there was not systematically higher pollution levels in the coastal environments (data not shown), which also is in line with our observations of detoxification systems. Interestingly, the multicopper oxidase and dioxygenase C families were the only protein families strongly related to the estimated pollution (Figure 5b). Overall, we observed that macrogeographic distribution might be more influential to the detoxification gene content than human-made pollutants (Figure 5 and Additional file 9: Figure S4), providing further indication that natural environmental factors have shaped the marine bacterial communities to a much larger extent than anthropogenic influence. These observations highlight the non-trivial relationships between pollutant selection pressure and how it shapes microbial communities in different environments. It is clear that to better understand these phenomena, future studies need to collect appropriate environmental data connected to the samples.

It should be stressed that many of these detoxification systems might not only be of use in handling man-made pollutants, but could equally well be involved in the detoxification of natural toxins in the open ocean. Several biotoxins are for instance produced by dinoflagellates and cyanobacteria [42, 43]. Marine bacteria like *Synechocystis* and *Synechococcus* can produce a number of toxic compounds and the increase in nutrients due to human activities, and consequently the increase of eutrophication, could enhance the possibility of harmful cyanobacterial bloom formation and result in higher levels of toxins. The effect of these marine biotoxins on the bacterial communities in the open ocean is presently not known. However, it has been proposed that marine bacteria, such as *Vibrio* and *Pseudomonas* spp., are capable of metabolizing biotoxins via oxidation reactions catalyzed by oxidases and peroxidases [44].

The large abundance of cyanobacteria could also explain the comparably high numbers of penicillin binding transpeptidases in the oceanic environment. Transpeptidases are present in cyanobacteria and are involved in biosynthesis and maintenance of bacterial peptidoglycan, required for proper formation of the cyanobacterial cell wall [45]. The peptidoglycan layer is also believed to be partially responsible for drought tolerance in cyanobacteria. Cyanobacterial transpeptidases have been suggested as a possible origin of beta-lactamase genes, providing resistance to e.g. penicillin [46], and have been found in e.g. most *Prochlorococcus* strains. It is difficult to address whether the identified penicillin binding transpeptidase sequences constitute a cell wall maintenance system and/or a detoxification system, as the sequence similarity between the two categories is high and the physiological mechanisms are not completely understood, especially in non-model organisms.

## Conclusions

We conclude that the ocean as a habitat poses severe restrictions to fitness by several environmental factors e.g. nutrient limitation and pH, while the selection pressure from toxicants in the marine environment seems to be comparably small. It is, however, important to note that the ocean does not only function as a habitat, but is also a huge dispersal matrix connecting habitable patches, also for bacterial species. Most of these opportunistic bacteria present in small quantities need to have a larger set of genes, as they not only cope with the harsh reality of the ocean, but also must be able to bloom or colonize once they find a suitable habitat. However, we here report that the majority of marine bacteria in the open ocean do not possess a large variety of detoxification systems and would be predicted to have a lower capacity to adapt to polluted environments. It will be interesting to, in the future, examine specific highly polluted marine sites over longer time-periods, as well as to functionally characterize novel detoxification systems in marine bacteria. This will be essential to provide good estimates of future anthropogenic consequences on microbial life in the sea and could provide crucial information as ecotoxicology continues to move into metagenomic community analysis.

## Methods

### Protein family selection

The Gene Ontology annotation [14] was used to select genes from the NCBI database related to detoxification mechanisms. The following nine GO-terms was used to extract genes: response to organic substance (0010033), response to molecule of fungal origin (0002238), response to molecule of bacterial origin (0002237), response to xenobiotic stimulus (0009410), response to antibiotic (0046677), response to drug (0042493), response to inorganic substance (0010035), response to toxin (0009636), and response to oxidative stress (0006979). To restrict this set to well characterized bacterial genes, only genes belonging to one of these categories that could also be found in *Escherichia coli* were selected, and their corresponding protein sequences were retrieved. To determine the Pfam (release 24) [12] protein domains present in these protein sequences, all sequences were scanned against the Pfam database of profile-HMMs using HMMER3 [15] This resulted in a list of 159 protein families (Additional file 2: Table S2) that was manually inspected and reduced into a list of 31 detoxification-related protein families (Table 1). In addition, the profile for RNA polymerase Rpb2, domain 6 (PF00562) was added to provide normalization information, and the profiles for DNA polymerase A (PF00476) and elongation factor TS (PF00889) were also added to the set for comparison to ubiquitously occurring protein families.

### Data mining and abundance data

The profile-HMM for each protein family in Table 1 was compared to the 6,028,191 proteins predicted from the GOS data set [19] using hmmsearch (part of HMMER3), applying the trusted cut-off threshold specified for each individual Pfam profile-HMM. Samples taken with different filter sizes (GS-01a, GS-01b and GS-25), as well as the sample indicated to contain post-sampling contamination (GS-00a) [32], were not excluded, as these samples are likely to show pronounced differences from the majority of the samples. The resulting number of sequences can be found under ”GOS Initial” in Table 1. Sequences producing significant matches were extracted, and scanned against the entire Pfam profile database. Only reciprocal hits, i.e. sequences that found the same profile as initially found them, were kept (”GOS Reciprocal” in Table 1). There does not seem to be a significant correlation between the profile lengths and the number of sequences found in the GOS data (Additional file 12: Figure S7). The absence of such a trend is important since that could be a potential source of positive bias for short Pfam profiles because of the fragmentary and short sequence nature of metagenomic sequence data. The same Pfam profiles and procedure were used to determine the number of detoxification genes in 835 sequenced and annotated bacterial genomes (Additional file 3). The average number of genes corresponding to each protein family can be found in Additional file 4: Table S3.

### Environmental distribution data

Environmental distribution data was retrieved using the GOS nucleotide read databases and their connected metadata [30], downloaded from CAMERA [31]. Additional estimates of chemical parameters and anthropogenic influence on 29 of the sample sites was derived from previous work with the GOS samples [33]. Only reads that overlapped with the ORF coding for a protein of interest were included in the sampling site data search. At each GOS sampling site, the reads reciprocally matching a certain protein family were counted. To allow for per genome estimates for the protein families, RNA polymerase Rpb2, domain 6 (PF00562), corresponding to the *rpoB* gene, was used for normalization. This protein family is almost ubiquitously occurring in a single copy in the sequenced bacterial genomes (Additional file 3; Additional file 4: Table S3; Additional file 5: Figure S1), making division by the number of found proteins containing a Pfam RNA_pol_Rpb2_6 domain a suitable normalization procedure. The *rpoB* normalized values were correlated to environmental data [33] and used for the principal component analysis (Additional file 10: Figure S5). Normalized values were then converted to log_2_-scale. Sites with more than three protein families finding no reads, and protein families with more than three sites containing no reads were removed. A small number of remaining zeros that could not be translated to log-scale were set to -10, and a heat map (Figure 2, top) was created using R [47], with the additional components marray [48], amap [49] and gplots [50]. Hierarchical clustering was done using Euclidean distances for both sampling sites and protein families. A similar heat map was generated for the gene content of the sequenced bacterial genomes (Additional file 5: Figure S1). However, these genome values were *not* normalized to the RNA-polymerase (PF00562) protein family, as that would have severely skewed the data in some cases. The detoxification gene counts for the bacterial genomes were used to normalize the GOS data and generate a heat map showing the abundance of the detoxification protein families compared to what would be expected from the bacterial genomes (Figure 2, bottom). In addition, a heat map consisting of only marine bacteria was constructed (Figure 1). Bacterial species were classified as marine depending on their presence in a recent study by Yooseph *et al.* [24], which analyzed the genomes of commonly found bacteria in the GOS data set. Finally, each abundance value from the GOS data was divided by the average count for the corresponding protein family in the marine bacterial genomes (Additional file 4: Table S3). The numbers produced were used to create a heat map showing the over- and underrepresentation relative to what would be expected from the marine bacterial genomes (Additional file 13: Figure S8). In cases where zero instances of a protein family were found in the genomes, GOS data was instead normalized using the value 0.01 (data for all protein families from each step can be found in Additional file 14).

To test whether our set of protein families was representative of marine bacterial detoxification systems in general, we subjected six detoxification families known to be involved in detoxification, but not included in our *E. coli*-derived data set (Additional file 8: Table S4), to the same procedure with HMMER searches and normalization as above (Figure 3).

### Availability of supporting data

The data sets supporting the results of this article are included within the article and its additional files.

## Electronic supplementary material

Additional file 1: Table S1: GO terms used to select the detoxification protein families. (XLSX 45 KB)

Additional file 2: Table S2: The 159 detoxification Pfam profile-HMMs considered after *E. coli* filtering. (XLS 42 KB)

Additional file 3: **Excel file containing the results of the protein family searches in the 835 bacterial genomes.** (XLSX 890 KB)

Additional file 4: Table S3: The average detoxification protein content of all bacterial genomes, all marine genomes, and the most common marine genomes. (XLSX 40 KB)

Additional file 5: Figure S1: Occurrence of detoxification protein families in all investigated 835 bacterial genomes. No gene found (grey), 1 gene per genome (black) and greater than 1 gene per genome (red). (PDF 430 KB)

Additional file 6: Figure S2.: Correlation between median and average values. Median and average numbers for copy number of the detoxification protein families in 835 bacterial genomes. (PDF 46 KB)

Additional file 7: Figure S3: Number of detoxification genes found in the GOS data. Numbers are compared to what would be expected from the average in 835 fully sequenced bacterial genomes. (PDF 35 KB)

Additional file 8: Table S4: Genome and GOS analysis corresponding to Table 1 for six additional detoxification protein families not included in our *E. coli*-based approach. (XLSX 35 KB)

Additional file 9: Figure S4: Abundance of detoxification proteins across macrogeographic locations. Comparison of the relative abundance of the detoxification proteins between the three geographic locations suggested by Patel *et al.* [33]: North-Atlantic (blue bars), Mid-Atlantic (red bars) and Pacific (green bars). Bars indicate relative abundance in relation to the protein family abundance in the North Atlantic sites (fixed to one) and only proteins that exhibited a significant difference in relative abundance between locations are displayed. Brackets indicate which comparisons that were significant; black brackets (p < 0.01) and grey brackets (p < 0.05). (PDF 36 KB)

Additional file 10: Figure S5.: Principal component analysis of correlated protein families and environmental features. Protein families are color coded according to category: red – oxidative stress, blue – metal resistance, green – transporters, purple – other detoxification systems, grey – control proteins. Environmental features are represented in black. (PDF 46 KB)

Additional file 11: Figure S6: Sample sites clustered by estimated pollution and shipping. The sites classified as polluted are marked by red. All other sites were classified as non-polluted (blue). Pollution and shipping data were estimated by Patel *et al.* [33]. (PDF 43 KB)

Additional file 12: Figure S7: Number of sequences matching to each of the initial 159 Pfam profile-HMMs. Numbers are plotted against the length of the Pfam profile. No significant correlation between length and matched sequences was observed. (PDF 63 KB)

Additional file 13: Figure S8: Distribution of detoxification protein families in the metagenomic GOS data. Normalized to the average gene content of all 61 marine species/strains (as listed in Figure 1) included in our survey of 835 bacterial genomes. (PDF 22 KB)

Additional file 14: **Excel file containing the results of the protein family searches from the GOS sampling sites.** (XLSX 452 KB)
