# Supplementary figures and images for: Metagenomics reveals that detoxification systems are underrepresented in marine bacterial communities
Source: BMC Genomics. 2014 Sep 1;15(1):749. doi: 10.1186/1471-2164-15-749 (PMC4161860; doi:10.1186/1471-2164-15-749)

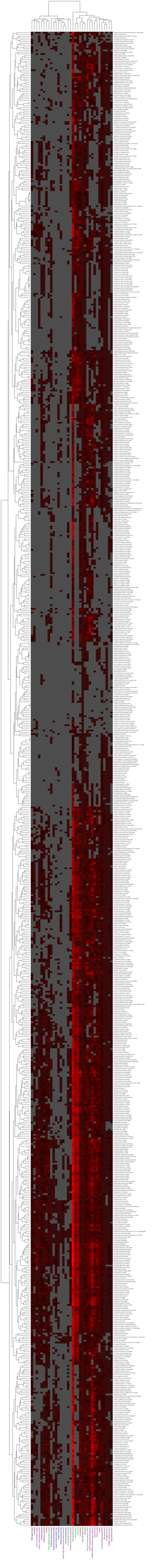

Supplement: Supplementary file 5 — Additional file 5: Figure S1: Occurrence of detoxification protein families in all investigated 835 bacterial genomes. No gene found (grey), 1 gene per genome (black) and greater than 1 gene per genome (red). (PDF 430 KB) [file 12864_2014_6429_MOESM5_ESM.pdf]

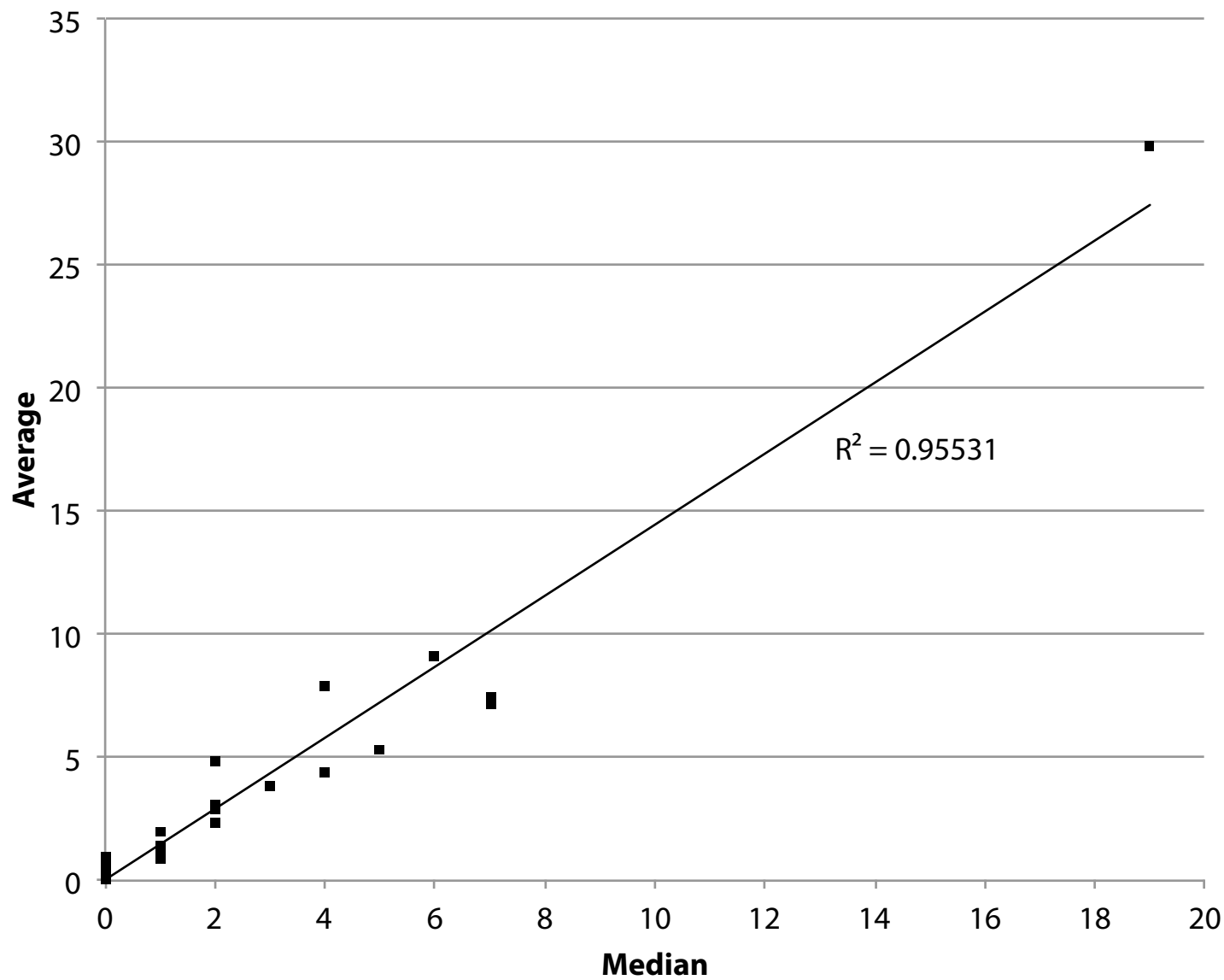

Supplement: Supplementary file 6 — Additional file 6: Figure S2.: Correlation between median and average values. Median and average numbers for copy number of the detoxification protein families in 835 bacterial genomes. (PDF 46 KB) [file 12864_2014_6429_MOESM6_ESM.pdf]

# Found genes per expected number of genes

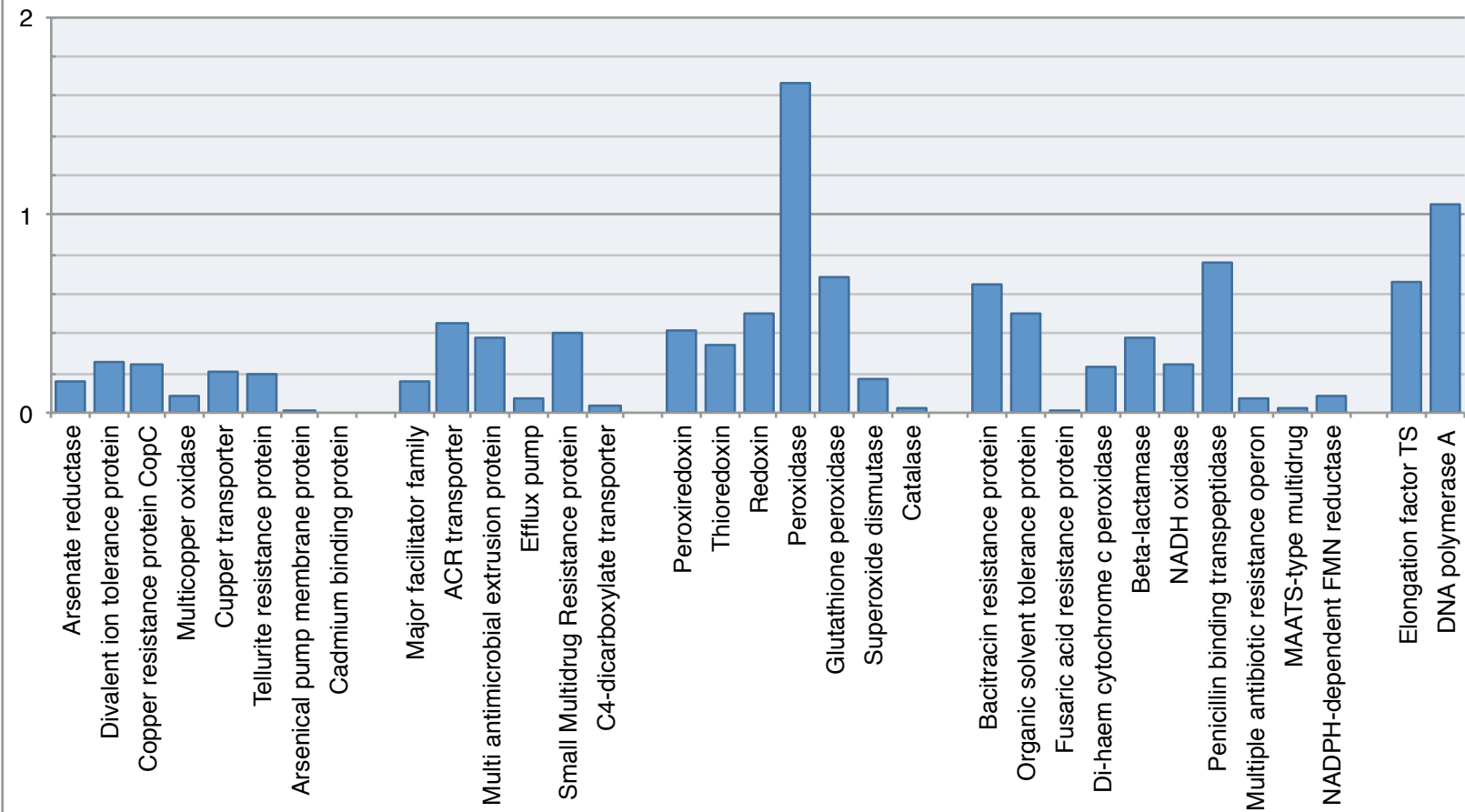

Supplement: Supplementary file 7 — Additional file 7: Figure S3: Number of detoxification genes found in the GOS data. Numbers are compared to what would be expected from the average in 835 fully sequenced bacterial genomes. (PDF 35 KB) [file 12864_2014_6429_MOESM7_ESM.pdf]

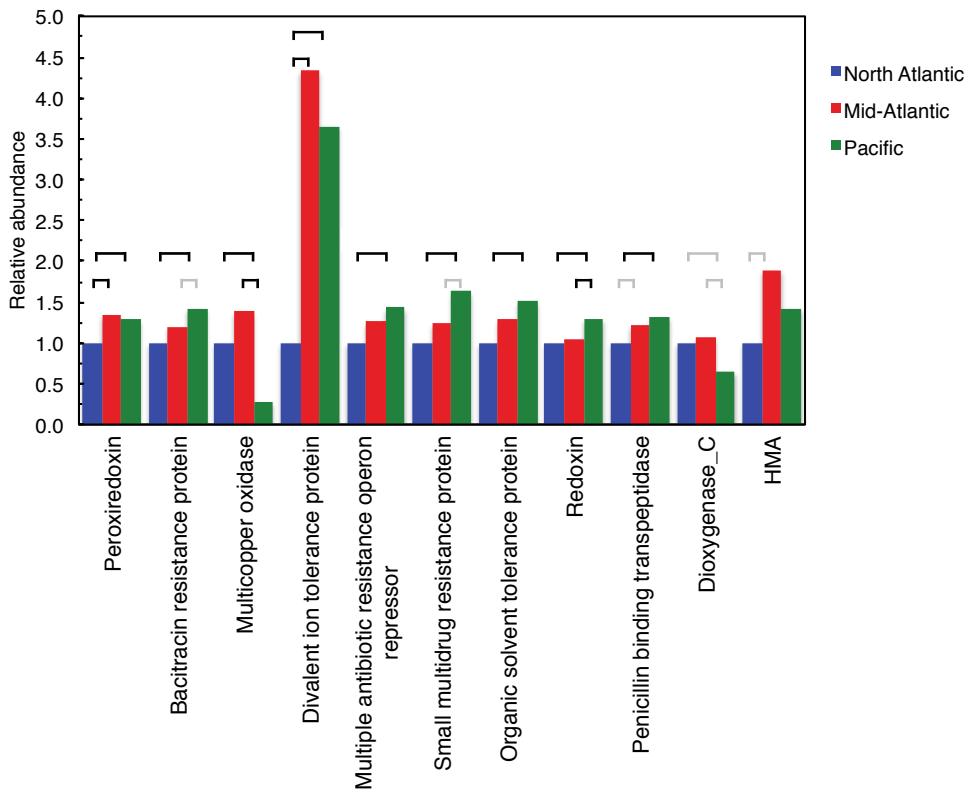

Supplement: Supplementary file 9 — Additional file 9: Figure S4: Abundance of detoxification proteins across macrogeographic locations. Comparison of the relative abundance of the detoxification proteins between the three geographic locations suggested by Patel et al. [33]: North-Atlantic (blue bars), Mid-Atlantic (red bars) and Pacific (green bars). Bars indicate relative abundance in relation to the protein family abundance in the North Atlantic sites (fixed to one) and only proteins that exhibited a significant difference in relative abundance between locations are displayed. Brackets indicate which comparisons that were significant; black brackets (p < 0.01) and grey brackets (p < 0.05). (PDF 36 KB) [file 12864_2014_6429_MOESM9_ESM.pdf]

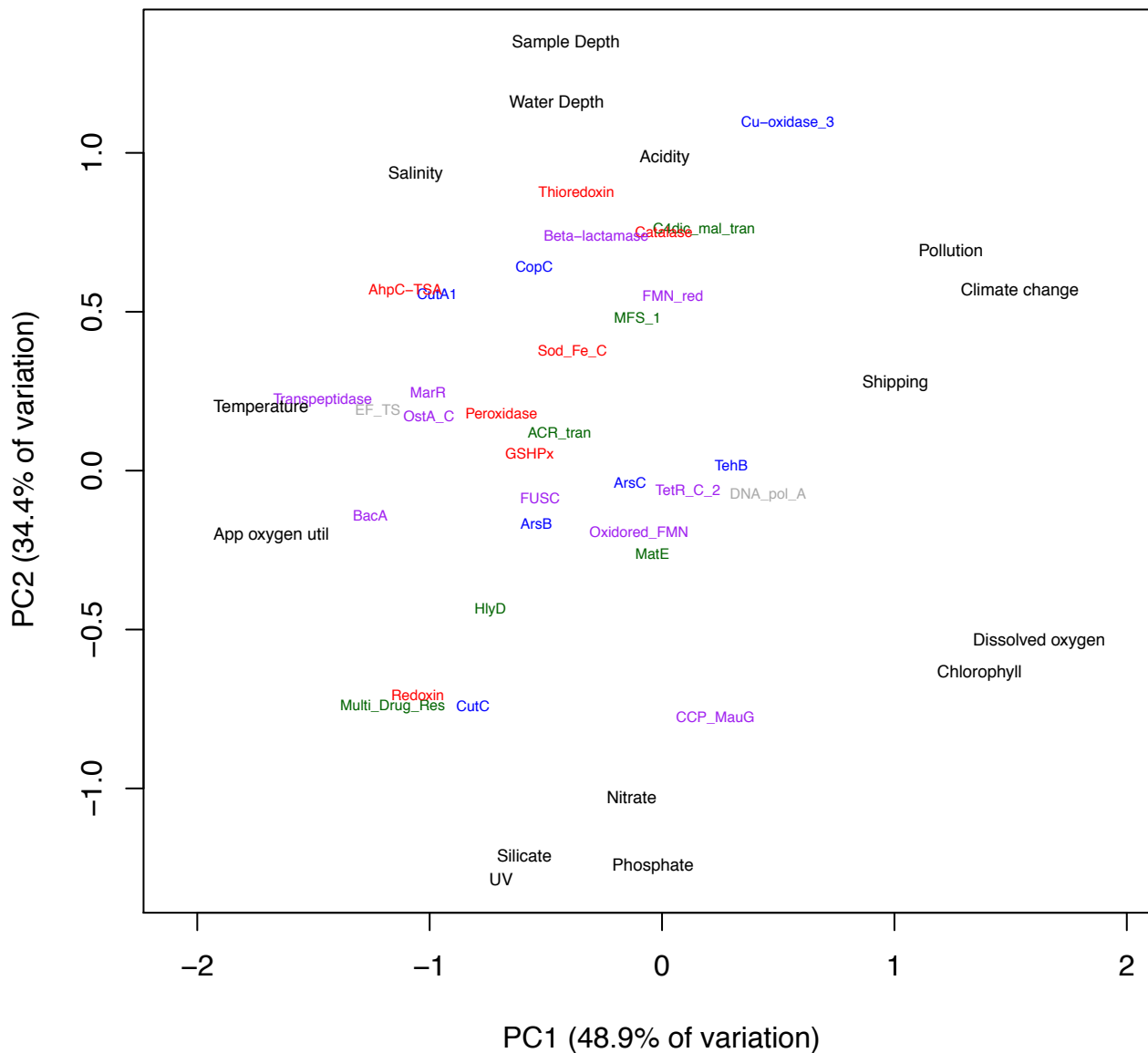

Supplement: Supplementary file 10 — Additional file 10: Figure S5.: Principal component analysis of correlated protein families and environmental features. Protein families are color coded according to category: red – oxidative stress, blue – metal resistance, green – transporters, purple – other detoxification systems, grey – control proteins. Environmental features are represented in black. (PDF 46 KB) [file 12864_2014_6429_MOESM10_ESM.pdf]

# Color Key and Histogram

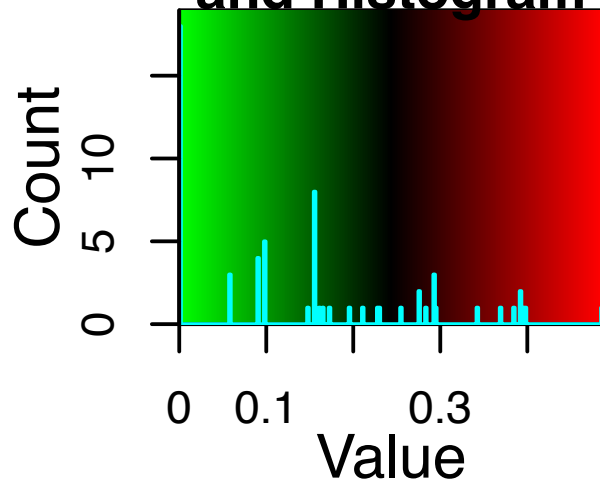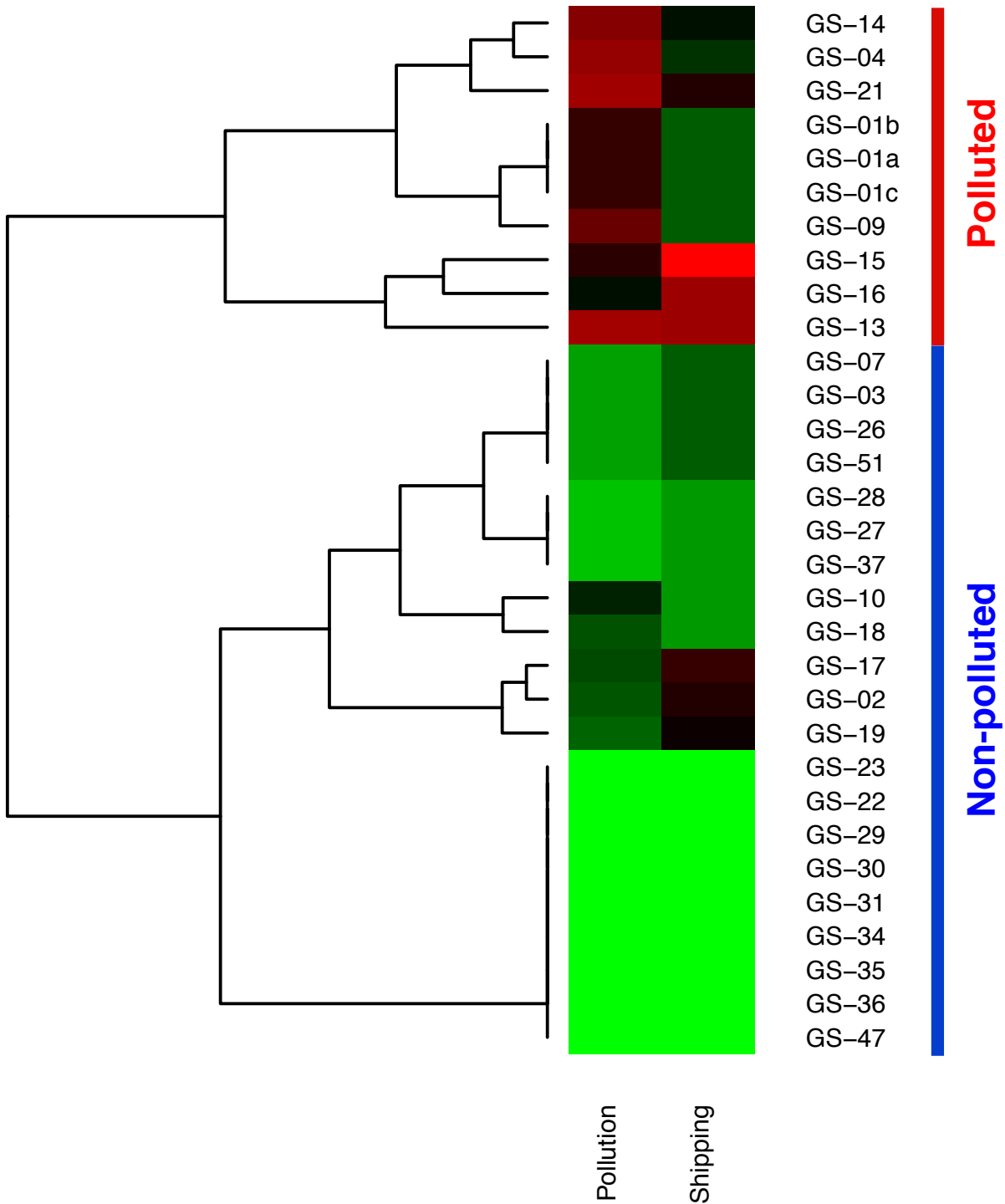

Supplement: Supplementary file 11 — Additional file 11: Figure S6: Sample sites clustered by estimated pollution and shipping. The sites classified as polluted are marked by red. All other sites were classified as non-polluted (blue). Pollution and shipping data were estimated by Patel et al. [33]. (PDF 43 KB) [file 12864_2014_6429_MOESM11_ESM.pdf]

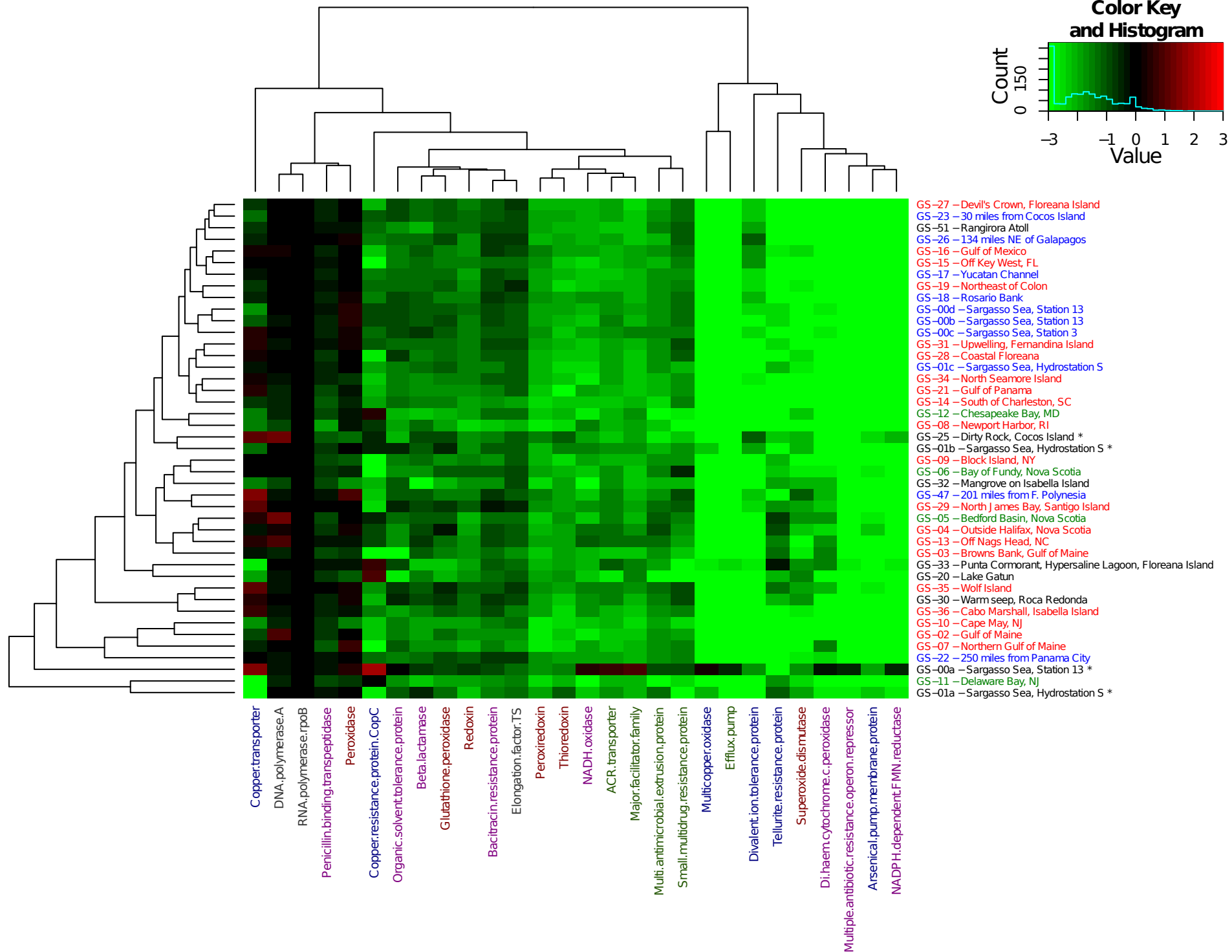

Supplement: Supplementary file 12 — Additional file 12: Figure S7: Number of sequences matching to each of the initial 159 Pfam profile-HMMs. Numbers are plotted against the length of the Pfam profile. No significant correlation between length and matched sequences was observed. (PDF 63 KB) [file 12864_2014_6429_MOESM12_ESM.pdf]

Profile length versus found sequences

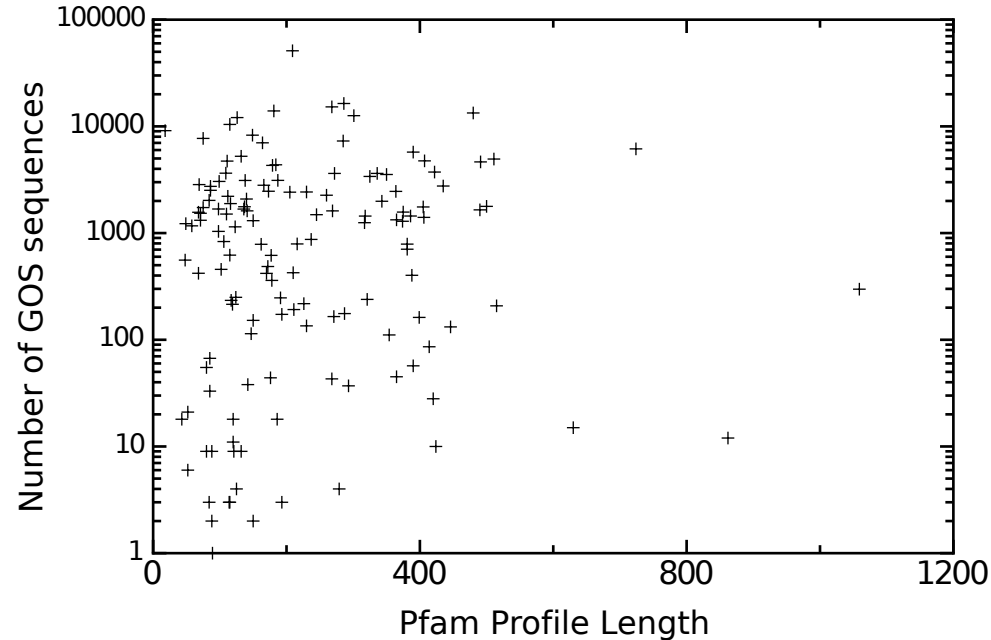

Supplement: Supplementary file 13 — Additional file 13: Figure S8: Distribution of detoxification protein families in the metagenomic GOS data. Normalized to the average gene content of all 61 marine species/strains (as listed in Figure 1) included in our survey of 835 bacterial genomes. (PDF 22 KB) [file 12864_2014_6429_MOESM13_ESM.pdf]
